# Could Systemic Inflammatory Index Predict Diabetic Kidney Injury in Type 2 Diabetes Mellitus?

**DOI:** 10.3390/diagnostics13122063

**Published:** 2023-06-14

**Authors:** Tuba Taslamacioglu Duman, Feyza Nihal Ozkul, Buse Balci

**Affiliations:** Department of Internal Medicine, Abant Izzet Baysal University Hospital, 14200 Bolu, Turkey; feyzanihalozkul41@gmail.com (F.N.O.); busebalcii@hotmail.com (B.B.)

**Keywords:** diabetic kidney injury, inflammation, systemic inflammatory index

## Abstract

Background: The systemic inflammatory index (SII) is a new inflammatory marker that has been the subject of various studies in diseases with chronic inflammation. Diabetic nephropathy is a disease associated with chronic inflammation. We aimed to evaluate the relationship between SII and diabetic nephropathy. Methods: Patients with diabetes who applied to our outpatient clinic were included in the study. Diabetic patients were divided into two groups: those with diabetic nephropathy and those without. In addition, healthy individuals who applied to our clinic for general check-ups during these dates were included as the control group. The SII values and other characteristics of the three study groups were compared. Results: The median SII value for those with DKI was 584 (178–4819); for those without DKI, it was 282 (64–618); and for the control group, it was 236 (77.5–617) (*p* < 0.001). SII was significantly and positively correlated with BMI, weight, blood glucose, HbA1c, CRP, and creatinine, and negatively correlated with the glomerular filtration rate (GFR) value. SII values higher than 336 have 75% sensitivity and 70% specificity in detecting DKI. Conclusion: The SII value can predict diabetic kidney injury in diabetics, and it can be used as an adjunctive diagnostic tool.

## 1. Introduction

Diabetes mellitus is a complex metabolic disorder marked by hyperglycemia and many complications that impact various organs throughout the body. The condition arises from an insufficient ability to utilize carbohydrates, proteins, and fats due to insulin deficiency or resistance [1]. Over 400 million individuals worldwide live with diabetes mellitus; experts predict this number will escalate to 600 million by 2035 [2]. Chronic complications of diabetes are divided into two main groups—micro and macrovascular. Microvascular complications are retinopathy, neuropathy, and nephropathy. A frequent complication of diabetes is diabetic nephropathy, also known as diabetic kidney injury. In diabetic patients, up to 20% may develop diabetic kidney disease, contributing significantly to morbidity and mortality due to end-stage renal failure [3]. With the increasing prevalence of diabetes, if treatment strategies for the prevention of diabetic nephropathy cannot be developed, the prevalence of diabetic nephropathy will increase in parallel with the increasing prevalence of diabetes. We know that approximately one-third of patients with diabetes develop diabetic nephropathy after latency periods, which can vary for several years [4]. Therefore, it is very important to screen for nephropathy in diabetic patients. In type 1 diabetes patients, this process is screened from the fifth year after diagnosis. In type 2 diabetes patients, albuminuria is immediately screened for nephropathy, like other complications of diabetes, as the effects of hyperglycemia can be seen before the diagnosis of diabetes, just like the iceberg effect.

Diabetic nephropathy is the most significant cause of end-stage renal disease. There is an increase in the prevalence of diabetic nephropathy globally, especially in developing countries [5]. Diabetes mellitus is responsible for many cases of end-stage renal disease in Western Europe and the US, with approximately 40% of individuals who require regular dialysis therapy also experiencing diabetes mellitus as an underlying cause [6]. In China, both the incidence and prevalence of diabetic nephropathy have increased significantly over the past decade, with the estimated number of diabetic patients with chronic kidney disease (CKD) reaching 24.3 million [7]. Therefore, tight glycemic control, strict blood pressure management, protein restriction, and discontinuation of smoking are essential in preventing microvascular complications in diabetic patients [8,9,10]. In addition to the duration of the disease, poorly controlled blood glucose is also an important contributor to the development of diabetic nephropathy in diabetic patients [4]. Long-term hyperglycemia is associated with diabetic complications in diabetic patients. In particular, it causes diabetic nephropathy, which is one of the microvascular complications of diabetes.

Unfortunately, the pathogenesis of diabetic nephropathy is quite complex and still not fully understood. Therefore, there is still weak therapeutic efficacy in the treatment of diabetic nephropathy. It has been shown that diabetic nephropathy can still progress to end-stage renal disease and increase mortality even with standard treatment, including tight blood sugar and blood pressure control. For all of these reasons, research to understand the mechanisms of the pathogenesis of diabetic nephropathy requires both the understanding of the cause and the development of new treatment strategies. Considering that oxidative stress, angiotensin II, and inflammatory processes—which are thought to play an important role in the pathogenesis of diabetic nephropathy—are important points in the development and progression of diabetic nephropathy, studies and new treatment strategies have focused on these issues.

It is known that oxidative stress plays a role in the pathogenesis of nephropathy in patients with hyperglycemia [11]. Activation of protein kinase C and nephropcation end products play a role in the pathogenesis of nephropathy. Nicotinamide deninedinucleotide plays a role in the pathogenesis of diabetic nephropathy due to increased renal oxidative stress production via phosphate oxidase. Increasing superoxide ion causes an increase in the urinary vascular endothelial growth factor, exacerbates oxidative stress, and contributes to the pathogenesis of diabetic nephropathy. Blocking the nicotinamide adeninedinucleotide phosphate oxidase enzyme with aposinin or another inhibitor has been associated with improvement in both albuminuria and glomerular sclerosis in patients with diabetic nephropathy [12].

Diabetic nephropathy is characterized by proteinuria due to glomerular damage and is accompanied by a decrease in the glomerular filtration rate. End-stage renal disease develops due to increased proteinuria and a decreased glomerular filtration rate. Diabetic nephropathy is the most common cause of both chronic renal failure and end-stage renal disease [13].

In the clinic, we provide diagnoses and follow-up care for diabetic nephropathy by evaluating the amount of albuminuria and proteinuria in urinalysis. Urinalysis is important in diabetic patients. In patients with nephropathy, the spot urine albumin/creatinine ratio, the spot urine protein/creatinine ratio, and 24-h urine albuminuria and proteinuria should be monitored. In patients with nephropathy, renoprotective medications are used in addition to controlling hyperglycemia to halt progression and treat nephropathy.

Tervaert et al. categorize diabetic nephropathy into four main glomerular lesions with a separate assessment for the degree of interstitial and vascular involvement [14]. Class I, glomerular basement membrane thickening: isolated glomerular basement membrane thickening and only mild, nonspecific changes that do not meet class II–IV criteria by light microscopy. Class II, mesangial enlargement, mild (IIa) or severe (IIb): glomeruli classified as mild or severe mesangial enlargement without nodular sclerosis (Kimmelstiel–Wilson lesions) or global glomerulosclerosis in more than 50% of the glomeruli. Class III, nodular sclerosis (Kimmelstiel–Wilson lesions): at least one glomeruli with nodular increase in the mesangial matrix (Kimmelstiel–Wilson) without the changes described in class IV. Class IV, advanced diabetic glomerulosclerosis: more than 50% global glomerulosclerosis with other clinical or pathological evidence that sclerosis is attributable to diabetic nephropathy [14].

The most common pathological changes identified in kidney biopsies of clinical diabetic nephropathy patients are glomerular lesions, particularly diffuse and nodular mesangial enlargement and glomerular basement membrane thickening. As the disease progresses, diffuse mesangial enlargement develops into nodular deposits of the mesangial matrix in the late stage of diabetic nephropathy. Also known as Kimmelstiel–Wilson nodules, these nodular lesions can be seen in approximately 25% of patients with advanced diabetic nephropathy [15,16,17].

Nodular lesions and diffuse lesions are the two stages of diabetic nephropathy. Patients with nodular diabetic glomerulosclerosis show more severe kidney injury, longer diabetic durations, and worse kidney prognosis compared with patients with diffuse mesangial enlargement. Diffuse mesangial enlargement, which develops in the fifth year from the onset of diabetes, is the earliest change that can be observed with light microscopy [14].

It is possible to classify diabetic kidney disease according to urinary albumin excretion and the degree of renal dysfunction. The period of microalbuminuria, in which there is very little albumin excretion, is considered the earliest stage of the disorder. However, the presence of microalbumin in the urine should be considered an early warning sign of serious kidney problems rather than an innocent finding. Indeed, treatments and approaches that reduce microalbuminuria have been reported to slow down and even reverse progressive kidney damage [18]. According to studies related to the onset and clinical course of diabetic nephropathy, it has been concluded that diabetic kidney disease begins as a result of the interaction of impaired metabolic and hemodynamic processes that are frequently encountered in the clinical course of diabetes [19]. Presumably, abnormalities in many metabolic and hemodynamic pathways seem to initiate renal damage either directly or by the effect of reactive oxygen derivatives acting at the cellular level due to reduced redox capacity. Metabolic disorders, increased reactive oxygen species, and hemodynamic abnormalities lead to activation or inhibition of many pathways at the immune mediator level by stimulating various gene transcription factors. As a clinical consequence, diabetic kidney disease develops, which is characterized by increased urinary albumin excretion and decreased kidney function [18].

Blocking the renin–angiotensin–aldosterone system, which is among the leading treatments to prevent or slow down diabetic renal disease, is based on either inhibiting the angiotensin converting enzyme or blocking the angiotensin receptor type 1. Angiotensin converting enzyme 2, which provides the conversion of angiotensin 1 to angiotensin 1–9 and angiotensin 2 to angiotensin 1–7, is a zinc-dependent enzyme and has been shown to be expressed in the heart and blood vessels, in addition to the kidney [19]. In animal experiments, ACE2 activity was shown to be reduced in the renal tubules after MLN-4760 (an ACE2 inhibitor molecule) was administered. Moreover, studies in diabetic mice have found a decrease in ACE2 expression in the renal tubules and, therefore, in renal angiotensin 1–7 levels [20]. Albuminuria decreases, blood pressure decreases, and there is no significant change in high GFR in animals administered ACE inhibitor; however, an increase in urine albumin level, an increase in blood pressure, and a decrease in hyperfiltration were found in animal experiments using ACE2 inhibitor [11]. Therefore, it is understood that the decrease in angiotensin 1–7 levels contributes to diabetic nephropathy.

One of the pathophysiological mechanisms in the development of diabetic nephropathy is excessive extracellular protein production in the kidney tissue. One of the agents responsible for the overproduction of extracellular matrix proteins and their accumulation in kidney tissue is transforming growth factor-β; its expression is increased by high plasma glucose, angiotensin 2, and some other fibrotic stimuli. In addition to these direct effects, transforming growth factor-β also indirectly contributes to diabetic nephropathy by increasing the levels of connective tissue growth factor [11].

Glomerular structural disorders are also thought to have an important role in the pathogenesis of diabetic nephropathy. In the early phase of the disease, the hyperfiltration phase and accompanying increased albumin excretion are characteristic features of diabetic nephropathy. The cells surrounding the glomerular capillaries are called podocytes. These cells are of neuroepithelial origin and show quite different characteristics. There are many proteins in the extensions of podocytes that surround the vessels. Nephrin, which is one of them, acts like a zipper and allows the passage of substances such as water and glucose, while the larger protein does not allow the passage of substances in the structure. Research shows that proteinuria and nephrin protein disorders play a role in the course of diabetic nephropathy. In diabetic patients, it has been shown that the foot protrusions of podocytes are retracted and flattened, resulting in thickening of the glomerular basement membrane. In animal experiments, it has been determined that there is a decrease in both the renal nephrin level and the number of protrusions in rats with diabetic nephropathy [21]. Not only diabetes mellitus but also hypertension affects the levels of nephrin. Recent studies suggested that nephrin levels show a gradual increase from birth to adulthood but were reduced in subjects with hypertension [22]. In diabetic animal studies, it was found that renal nephrin gene expression and nephrin levels decreased, but irbesartan reversed this decrease [21]. Similar to Valsartan, nephrin has been shown to increase nephrin gene expression and glomerular nephrin levels [23]. Studies investigating whether this effect is due to RAS blockade or the antihypertensive effects of these agents found that, although similar blood pressure reductions were obtained with amlodipine, there was no change in nephrin levels [23].

Understanding the key features of inflammatory mechanisms involved in the development and progression of diabetic nephropathy also allows for the identification of new potential targets and facilitates the design of innovative anti-inflammatory therapeutic strategies. Structural changes that occur in diabetic nephropathy can affect all renal compartments. Current literature suggests that both systemic and local renal inflammation play critical roles in the progression of diabetic nephropathy [24]. We know the nephropathy effect of inflammation caused by the effect of hyperglycemia. Several studies have shown that inflammation contributes to impaired kidney function. High-sensitivity C-reactive protein (hs-CRP) is a systemic inflammatory marker associated with the progression of diabetic nephropathy in patients with type 2 diabetes mellitus [25,26]. The CRP/albumin (CAR) ratio has also been associated with inflammation and nephropathy in diabetic patients [27]. Platelet/lymphocyte and neutrophil/lymphocyte ratios, which are inflammatory markers, have also been used in many studies to diagnose and predict the prognosis of microvascular complications of type 2 diabetes mellitus [28]. Parameters such as platelet/lymphocyte and neutrophil/lymphocyte ratios, and mean platelet volume/lymphocyte ratios are inflammatory markers derived from hemogram and have been the subject of studies on chronic inflammation diseases such as diabetes. Hemogram-derived inflammatory markers have been studied for a few decades in the medical literature. The first hemogram marker that was observed in inflammatory conditions was mean platelet volume. Many studies in the literature showed that inflammatory conditions were related to either increased or decreased serum levels of mean platelet volume. While diseases with a high grade of inflammation, such as infection [29] and rheumatoid arthritis [30], were associated with increased levels of mean platelet volume, decreased mean platelet volume was noted in conditions that were characterized by subtle inflammation. In the literature, inflammatory markers related to this hemogram have also been associated with diabetic nephropathy [31].

Another novel inflammatory marker has been developed by using hemogram markers, including neutrophil count, lymphocyte count, and platelet count. This new marker has been called the systemic inflammatory index (SII). The systemic inflammatory index is a novel inflammatory marker that has been studied for its potential to reflect local immune response and systemic inflammation. It was developed based on neutrophil, lymphocyte, and platelet counts; thus, the same inflammatory mechanisms that cause alteration in these cells may also cause an increase in SII levels. Cytokines either increase or decrease during inflammatory processes. For example, highly sensitive CRP increases and neuregulin-4 decreases during inflammation. This marker—which is derived from a test that can be performed anywhere, such as a hemogram, with cheap and rapid results—is valuable as an indicator of inflammation because it is calculated with neutrophils, lymphocytes, and platelets in the hemogram. The systemic inflammatory index is calculated with the following formula: (SII = platelet count × neutrophil count/lymphocyte count). It was first used by Hu et al. to predict the prognosis of patients with hepatocellular carcinoma [32]. The SII value has been the subject of many cancer-related studies after HCC. It has been proven to have a high predictive value in various tumors such as hepatocellular carcinoma, small cell lung cancer, ovarian cancer, esophageal cancer, colorectal cancer, and cervical cancer [32,33,34,35,36,37]. The SII value has also been studied as an indicator of inflammation in diabetic patients with depression [38]. SII is believed to provide a more accurate evaluation of the inflammatory status.

This study aimed to assess the potential of SII, an inflammatory marker, as a predictive biomarker for identifying kidney damage in diabetic nephropathy, a microvascular complication of diabetes.

## 2. Materials and Methods

This study includes patients diagnosed with type 2 diabetes mellitus who applied to the internal medicine clinics of Bolu Abant Izzet Baysal University Medical Faculty Hospital between March 2020 and December 2022. Diabetic patients are divided into two groups: those with diabetic nephropathy and those without. In addition, healthy individuals who applied to our clinic for general check-ups during these dates are included in the control group.

Patients diagnosed with type 2 diabetes mellitus who are 18 or older and healthy controls over 18 are included in the study. Excluded from the study are patients under 18; those diagnosed with type 1 diabetes mellitus; those with malignancies, liver failure, and autoimmune and rheumatic diseases; pregnant women; those with acute infections; those with diabetic feet; those who have recently undergone surgery; and those who refused to participate.

Patient data—such as age, gender, body mass index, and duration of diabetes—are recorded from the outpatient clinic records. Additionally, laboratory values such as complete blood count; kidney, thyroid, and liver function tests; serum albumin levels; fasting glucose; glycated hemoglobin (HbA1c); C-reactive protein; spot urine protein; spot urine microalbumin; and spot urine creatinine are recorded. The systemic immune-inflammation index (SII), calculated from the complete blood count (SII = platelet count × neutrophil count/lymphocyte count), is also recorded. The SII values and other characteristics of the three study groups are compared.

After data collection, the statistical program SPSS (SPSS 20.0 for Windows IBM Inc., Chicago, IL, USA) is used to analyze the data. The Kolmogorov–Smirnov test is used to assess normal distribution, while categorical variables are compared using the chi-square test. The Kruskal–Wallis test is used to evaluate whether there is a significant difference in median values between groups. Correlation analysis is performed with Pearson’s correlation test. Logistic regression analysis considering other confounding factors as well as SII is performed. ROC curve analysis is used to determine the sensitivity and specificity of SII in predicting diabetic nephropathy. A *p*-value of less than 0.05 will be considered statistically significant.

## 3. Results

There were 539 participants, of which 126 had type 2 diabetes mellitus with diabetic kidney injury, 227 had type 2 diabetes mellitus without diabetic kidney injury, and 186 were healthy controls. The median ages for each group were 59 (41–86) for those with DKI, 58 (29–76) for those without DKI, and 53 (18–76) for the control group. Age was found to be statistically different between each group (*p* < 0.001).

The participants with DKI consisted of 71 (56%) women and 59 (44%) men, while those without DKI included 134 (59%) women and 93 (41%) men. The healthy control group included 37 (20%) women and 149 (80%) men. Gender was significantly different between the groups (*p* < 0.001). Table 1 indicates the characteristics of the participants.

There were no differences in terms of diastolic blood pressure (*p* = 0.87), platelet count (*p* = 0.34), urea (*p* = 0.12), creatine (*p* = 0.24), LDL cholesterol (*p* = 0.35), and total cholesterol (*p* = 0.12) among each group. However, the BMI (*p* < 0.001), weight (*p* < 0.001), waist circumference (*p* < 0.001), systolic blood pressure (*p* = 0.02), white blood cell count (*p* < 0.001), lymphocyte count (*p* < 0.001), neutrophil count (*p* < 0.001), hemoglobin (*p* < 0.001), hematocrit (*p* < 0.001), blood glucose (*p* < 0.001), HbA1c (*p* < 0.00.), CRP (*p* < 0.001), serum albumin (*p* < 0.001), AST (*p* < 0.001), ALT (*p* < 0.001), and HDL cholesterol (*p* < 0.001) were found to be different, statistically. Table 2 and Table 3 show the values of the study groups.

The median SII value for those with DKI was 584 (178–4819); for those without DKI, it was 282 (64–618); and for the control group, it was 236 (77.5–617) (*p* < 0.001).

Smoking rates did not differ between the groups, but alcohol consumption varied significantly (*p* = 0.03). The rates of retinopathy and neuropathy were higher in the DKI group (*p* = 0.03 and *p* < 0.001, respectively).

Correlation analyses revealed that SII was significantly and positively correlated with BMI (r = 0.14, *p* = 0.045), weight (r = 0.29, *p* = 0.04), blood glucose (r= 0.12, *p* = 0.007), HbA1c (r = 0.14, *p* = 0.002), CRP (r = 0.35, *p* < 0.001), and creatinine (r = 0.12, *p* = 0.004). However, the glomerular filtration rate (GFR) value was significantly and negatively correlated (r = −0.26, *p* < 0.001) with SII.

**Figure 1:**
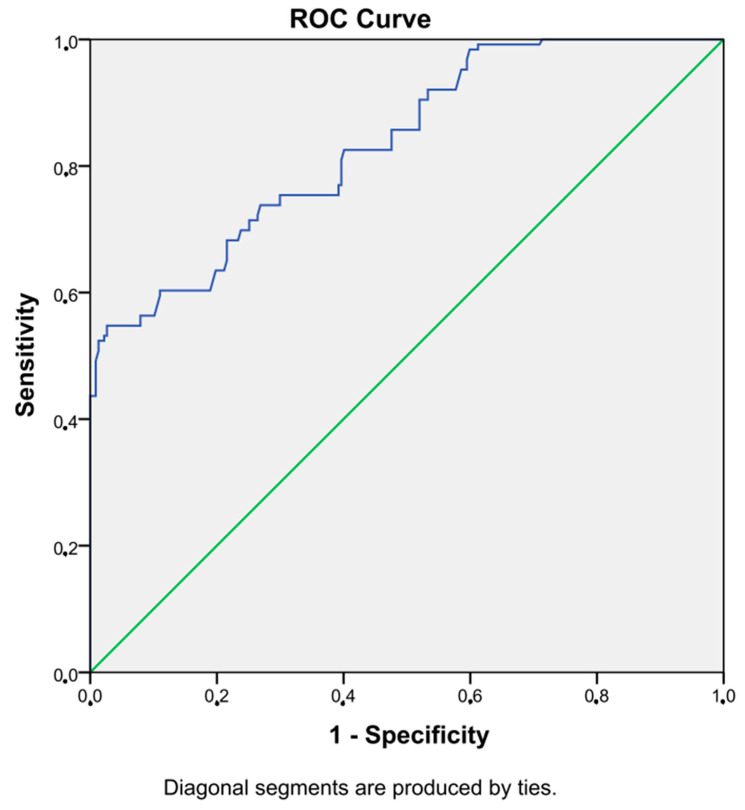
ROC curve of SII.

An SII value higher than 336 showed DKI with 75% sensitivity and 70% specificity (AUC = 0.83, *p* < 0.001, 95% CI = 0.79–0.88). Figure 1 shows the ROC curve of SII.

A binary logistic regression analysis considering age, gender, BMI, GFR, triglyceride, and SII revealed that SII was an independent risk factor for DKI (*p* < 0.001, OR = 1.29, 95% CI = 1.01–1.42).

## 4. Discussion

In our current study, we showed that the SII value is an effective marker to predict DKI in diabetes, and it could be used as a diagnostic tool in DKI. We also found that SII is significantly and positively correlated with BMI, weight, blood glucose, HbA1c, CRP, and creatinine. In addition, the glomerular filtration rate (GFR) value was significantly and negatively correlated with SII. We also showed that the SII value has 75% sensitivity and 70% specificity in detecting DKI.

SII is a novel inflammatory index which is calculated using neutrophils, lymphocytes, and platelets; all of these cells are important in inflammation [32]. Thus, this formula increases the importance of the SII value in inflammatory conditions. In the literature, SII has been studied in some cancers such as hepatocellular carcinoma, small lung cancer, epithelial ovarian cancer, esophageal squamous cell carcinoma, colorectal carcinoma, and cervical carcinoma, as well as some inflammatory conditions such as diabetes and diabetic microvascular complications [32,33,34,35,36,37,38,39]. Cancer is associated with increased inflammatory burden as is diabetic kidney injury. Therefore, increased SII values in diabetic kidney injury as presented in our study are a consistent finding in literature data.

As is well established, diabetes and its microvascular complications are considered as inflammatory diseases. Diabetic kidney injury, which is a microvascular complication of diabetes, is associated with chronic and low-grade inflammation. Microvascular injury is a principal factor of both systemic and local kidney inflammation, with the involvement of inflammatory cells such as neutrophils, monocytes, lymphocytes, and platelet, which are related to the development of diabetic kidney injury in patients with diabetes [40,41,42,43].

Neutrophils in peripheral blood are especially connected to the pathogenesis of diabetic kidney injury, as hyperglycemia induces an increase in the number of circulating neutrophils. Neutrophils migrate through chemokines to the glomerular basement membrane injury site. Thus, the inflammatory cascade begins [44].

Leukocytes also play an important role in renal disease progression. Leukocytes affect the kidney, including inflammatory mechanisms independent of infection, causing proteolytic and oxidative damage to the mesangial cells [45]. Activated leukocytes secrete many kinds of cytokines and transcription factors that have a crucial role in inflammation—including TNF-, TNF-B, interleukin-1, and transforming growth factor—thereby contributing to glomerulosclerosis [45,46,47,48,49]. Macrophages and lymphocytes are also effective in the earliest and other progressive stages of diabetic nephropathy [50]. Thus, it is understood that chronic inflammation and hyperglycemia play an important role in the development of diabetic kidney injury. Both neutrophils and lymphocytes contribute to SII; therefore, elevated SII levels in diabetic kidney injury are prominent in the present study. The findings of this study may have significant clinical implications for the timely identification and management of diabetic nephropathy in patients with type 2 diabetes mellitus.

A study conducted in Turkey observed serum uric acid levels as a potential predictor of diabetic nephropathy. The study revealed that high serum uric acid levels could be related to deteriorated kidney functions and increased albumin excretion in diabetic nephropathy patients. In addition, serum uric acid was positively correlated with daily albuminuria in that study [51].

Other inflammatory markers derived from a hemogram, such as neutrophil lymphocyte ratio (NLR), and those derived from the C-reactive protein, such as the C-reactive protein/albumin rate (CAR), are also found to be associated with DKI [27,52]. For example, the authors found that increased serum levels of the C-reactive protein/albumin rate have been associated with renal damage in diabetics with nephropathy in the CARE TIME study [27]. In this study, the C-reactive protein/albumin rate was also correlated with glycated hemoglobin and serum creatinine levels.

C-reactive protein is related to insulin resistance and hyperglycemia, and hyperglycemia and elevated C-reactive protein levels are also associated with the development of diabetic nephropathy. It is also well known that serum albumin levels have been defined as negative markers of inflammation. Thus, the C-reactive protein/albumin rate is elevated in inflammatory conditions like diabetes and its microvascular complications.

Poorly controlled type 2 diabetes and obesity are also associated with inflammatory markers like the neutrophil lymphocyte ratio and the C-reactive protein/albumin rate [26,27,52]. Another inflammatory marker in this regard is the monocyte–to–lymphocyte ratio. The MADKID study observed diabetic patients with and without diabetic kidney injury. The study consisted of 212 subjects from nephropathy and without nephropathy groups, and the authors found that the monocyte–to–lymphocyte ratio significantly increased in diabetic patients with diabetic kidney injury compared to subjects without diabetic kidney disease [53]. Another study evaluated 162 subjects with and without diabetic nephropathy. The authors found that a novel inflammatory marker, the mean platelet volume–to–lymphocyte count ratio, was significantly elevated in diabetic patients with nephropathy compared to that in diabetics without nephropathy. Moreover, the mean platelet volume–to–lymphocyte count ratio had considerably high sensitivity and specificity in selecting diabetic patients with kidney damage [31].

The elevated SII value in diabetic kidney injury in the present study is not surprising since the SII value is also an inflammatory marker. Accordingly, in our study, increased SII values, and thus increased inflammation, are positively correlated with CRP, BMI, weight, blood glucose, HbA1c, and creatinine, and negatively correlated with the glomerular filtration rate (GFR), similar to other findings in the literature.

Our study had some limitations. The retrospective study design and relatively small study population are two of these limitations. Another limitation is the single-center nature of the work.

## 5. Conclusions

In conclusion, we suggest that the SII value could be evaluated in diabetic patients suspected of DKI, as an adjunctive diagnostic tool. There is a great need for markers such as the SII value in the evaluation of diabetic nephropathy. The pathogenesis of diabetic nephropathy, which is still unexplained, is becoming a global problem, increasing day by day. There is a need for new research and new treatment strategies.

## Figures and Tables

**Table 1:** Characteristics of patients.

	DKI Present *n* (%)	DKI Absent *n* (%)	Healthy *n* (%)	*p* Value
Gender				
Female	71 (56.3%)	134 (59.0%)	37 (19.9%)	**<0.001**
Male	55 (43.7%)	93 (41.0%)	149 (80.1%)	
Smoking				
User	25 (20.0%)	37 (16.3%)	17 (10.7%)	0.088
Non-user	100 (80.0%)	190 (83.7%)	142 (89.3%)	
Alcohol				
Consumer	6 (4.8%)	2 (0.9%)	0 (0.0%)	**0.003**
Non-consumer	120 (95.2%)	225 (99.1%)	159 (100.0%)	
Retinopathy				
Present	17 (13.5%)	15 (6.6%)	-	0.031
Absent	109 (96.5%)	212 (93.4%)		
Neuropathy				
Present	86 (68.3%)	52 (22.9%)	-	**<0.001**
Absent	40 (31.7%)	175 (77.1%)		

**Table 2:** General characteristics of the study cohort.

	DKI Present	DKI Absent	Healthy	*p* Value *
	Median (Min–Max)			
Age (years)	59 (41.86)	58 (29.76)	53 (18.76)	**<0.001**
Height (cm)	1.59 (1.46–1.81)	1.61 (1.45–1.85)	1.68 (1.52–1.87)	**<0.001**
Weight (kg)	78 (48–106)	86 (59–117)	187.3 (55–136)	**<0.001**
BMI (kg/m^2^)	29.1 (16.6–43.5)	32 (21.9–46.1)	27.7 (18.3–49.4)	**<0.001**
Waist Circumference (cm)	102 (75–126)	107 (82–104)	98 (65–144)	**<0.001**
Systolic Blood Pressure (mmHg)	120 (90–180)	130 (100–180)	120 (90–180)	**0.02**
Diastolic Blood Pressure (mmHg)	80 (50–110)	80 (60–100)	80 (50–105)	0.867

* Kruskal–Wallis test was performed. Significant *p* values were expressed bold characters.

**Table 3:** Laboratory parameters of the participants.

	DKI Present	DKI Absent	Healthy	*p* Value *
Median (Min–Max)				
WBC (mm^3^/count)	6.240 (3.360–10.800)	5.050 (2.500–13.700)	5.500 (1.240–14.100)	**<0.001**
Plt (mm^3^/count)	251.000 (92.600–418.000)	229.000 (150.000–915.000)	239.000 (151.000–374.000)	**<0.001**
Neu (mm^3^/count)	2.085 (356–7.170)	2.470 (980–109.000)	3.200 (1.000–8.330)	**<0.001**
Lym (mm^3^/count)	1980 (356–3900)	1950 (1120–5010)	2100 (833–4510)	**<0.001**
Hb (g/dL)	13.1 (9.8–16.2)	13.3 (10.2–17.9)	14 (8.8–17.1)	**<0.001**
Hct (%)	38.8 (29.7–48.5)	38.1 (31–51.3)	42 (27.3–51.2)	**<0.001**
Fasting Blood Glucose (mg/dL)	186 (86–565)	143 (92–514)	93 (69–118)	**<0.001**
HbA1c (mmoL/dL)	9 (6.4–16.5)	7.6 (5.1–16)	5.4 (5.2–6.8)	**<0.001**
CRP (mg/dL)	8.1 (0.9–45)	3.4 (0.01–22)	2.4 (0.1–11.9)	**<0.001**
Urea (mg/dL)	30 (17.58)	32 (13–57.8)	28 (13–62)	0.124
Creatine (mg/dL)	0.8 (0.63–1.38)	0.78 (0.66–1.87)	0.8 (0.4–2.7)	0.235
GFR (mL/min)	96.3 (39.14–150.8)	111.5 (51.2–187.2)	115.2 (88.4–208)	**0.001**
Uric Acid (mg/dL)	5.5 (3.2–10)	5.5 (2.4–9.6)	5.7 (2.5–10.4)	0.071
Serum Albumin (g/L)	4.3 (3.5–5.1)	4.4 (3.9–4.9)	4.5 (3.8–5.1)	**0.001**
AST (U/L)	16 (11–39)	19 (8–39)	18 (9–31)	**<0.001**
ALT(U/L)	18 (8–64)	23 (6–94)	19 (6–58)	**<0.001**
Total Cholesterol (mg/dL)	208 (107–318)	198 (92–325)	200 (114–290)	0.124
Triglyceride (mg/dL)	176 (47–411)	153 (50–1050)	134 (52–680)	**0.013**
LDL (mg/dL)	126 (42.3–200)	119 (42–244)	115 (49–192)	0.352
HDL (mg/dL)	49.8 (26.8–86.6)	44 (25.5–61)	47 (20.6–85.2)	**<0.001**
SII	584 (178.4–4819)	282.5 (64.3–618.4)	236 (77.4–616.7)	**<0.001**

* Kruskal–Wallis test was performed. Significant *p* values were expressed bold characters.

## Data Availability

Datasets associated with this study are available from the corresponding author on reasonable request.
